# Gut microbial CAZymes markers for depression

**DOI:** 10.1038/s41398-024-02850-x

**Published:** 2024-03-05

**Authors:** Peijun Xie, Xingyu Zhou, Yifan Li, Jing Wu, Hanping Zhang, Yu Huang, Xunmin Tan, Lu Wen, Oluwatayo Israel Olasunkanmi, Jingjing Zhou, Zuoli Sun, Min Liu, Guofu Zhang, Ying Wang, Peng Xie, Jian Yang, Peng Zheng

**Affiliations:** 1 Department of Neurology, The First Affiliated Hospital of Chongqing Medical University, Chongqing, China; 2 NHC Key Laboratory of Diagnosis and Treatment on Brain Functional Diseases, The First Affiliated Hospital of Chongqing Medical University, Chongqing, China; 3 Beijing Key Laboratory of Mental Disorders, National Clinical Research Center for Mental Disorders & National Center for Mental Disorders, Beijing Anding Hospital, Capital Medical University, Beijing, China; 4 Advanced Innovation Center for Human Brain Protection, Capital Medical University, Beijing, China; 5 Department of Rehabilitation, The Second Affiliated Hospital of Chongqing Medical University, Chongqing, 400010 China; 6 Institute for Brain Science and Disease, Chongqing Medical University, Chongqing, China; 7 Key Laboratory of Major Brain Disease and Aging Research (Ministry of Education), Chongqing Medical University, Chongqing, China

**Keywords:** Psychology, Biomarkers

## Abstract

Major depressive disorder (MDD) is a serious mental illness, characterized by disturbances of gut microbiome, it is required to further explore how the carbohydrate-active enzymes (CAZymes) were changed in MDD. Here, using the metagenomic data from patients with MDD (*n* = 118) and heath controls (HC, *n* = 118), we found that the whole CAZymes signatures of MDD were significantly discriminated from that in HC. α-diversity indexes of the two groups were also significantly different. The patients with MDD were characterized by enriched Glycoside Hydrolases (GHs) and Polysaccharide Lyases (PLs) relative to HC. A panel of makers composed of 9 CAZymes mainly belonging to GHs enabled to discriminate the patients with MDD and HC with AUC of 0.824. In addition, this marker panel could classify blinded test samples from the two groups with an AUC of 0.736. Moreover, we found that baseline 4 CAZymes levels also could predict the antidepressant efficacy after adjusted confounding factors and times of depressive episode. Our findings showed that MDD was associated with disturbances of gut CAZymes, which may help to develop diagnostic and predictive tools for depression.

## Introduction

Major depressive disorder (MDD) is a serious and disabling mental illness, affecting up to 15% of the general population [1]. Currently, there are many theories about the pathogenesis of MDD, but they still can’t reveal its whole picture. Current antidepressants developed based on the existing theories can only make 30–50% of patients achieve clinical remission [2, 3]. In addition, clinically, the diagnosis of depression is mainly based on its clinical symptoms and scales. In developing countries, due to lack of professional psychiatrists, and the missed diagnosis and misdiagnosis rate of MDD is still high [4]. Therefore, it is of great clinical value to reveal the new potential molecular mechanisms of depression and identify the potential biomarkers for MDD.

Gut microbiome, an integral part of host biology, harbors about 90 families of carbohydrate-active enzymes (CAZymes) in healthy humans [5–7]. By contrast, there are only 4 enzyme families of the human genome, which only degrades starch, trehalose, and sugars [8]. In human, the more complex the structure of carbohydrates, the more diverse the enzyme system is needed. Thus, the gut microbiome enables to decompose complex fibers, and convert sugar units into energy to maintain the host’s health [9, 10]. In recent years, increasing clinical and basic studies showed that gut microbiome enables to substantially modulate the brain function and behaviors through microbiome-gut-brain (MGB) axis [11]. Meanwhile, disturbances of gut microbiome were implicated with development of mental diseases, such as autism, anxiety and schizophrenia [12–14].

In regarding of MDD, similar phenomenon was also observed. For examples, using the well-matched clinical samples, we found that patients with MDD were characterized by enriched the Bacteroidetes species relative to HC [15], showing a disease specific manner relative to patients with bipolar disorder [16]. As Bacteroidetes have more CAZymes-encoding genes [8], we speculate that patients with MDD may be accompanied by alternations of gut microbial CAZymes.

To test this hypothesis, using the whole-genome shotgun metagenomics method, we compared the microbial CAZymes signatures of patients with MDD and HC. Multivariate statistical method was used to explore the differences of gut microbial CAZymes signatures between the two groups. Meanwhile, the diagnostic and predictive values of candidate CAZymes biomarkers were also evaluated in MDD.

## Methods

### Ethics and participants

Ethical approval was obtained from the Human Research and Ethics Committee of Beijing Anding Hospital (#2017–24), Capital Medical University (China) aligning with the Declaration of Helsinki. The study design complied with all relevant ethical regulations, and all participants provided written informed consent.

### Sample collection

Totally, 311 individuals (aged 18–50 years old) were included in this study, including 155 HC and 156 patients with MDD(Table. S1). These samples were obtained from previous metagenomic cohort [15]. According to the Chinese version of the Mini-International Neuropsychiatric Interview (MINI), all MDD patients met the standard of DSM-IV. The Hamilton Depression Rating Scale (HAMD) and quick inventory of depressive symptomatology (QIDS) were used to evaluate the depressive severity and manic symptoms of MDD and BD. Patients with HAMD score of 0–3 is defined as normal, 4–7 as marginal, 8–15 as mild, 16–26 as moderate, and >27 as severe [17]. A QIDS score of <6 is defined as normal, 6–10 as mild, 11–15 as moderate, and >16 as severe [18]. Participants who used anti-depressant before collection were excluded in this study. Other exclusion criteria included: (1) History of other mental disorders; (2) suffering from chronic inflammatory disease, diabetes, cardiovascular disease, thyroid disease or cancer; (3) alcohol or drug abuse, acute poisoning; (4) pregnancy or breastfeeding; (5) current administration of antibiotics or long-term use of probiotics.

### DNA extraction and sequencing

Fecal samples were stored at −20 °C immediately after collection in the recruitment center, and then wrapped in dry ice and transported to the cryopreservation center. The total genomic DNA of fecal samples was extracted by E.Z.N.A.® Soil DNA Kit (Omega Bio-tek, Norcross, GA, USA) according to the manufacturer’s instructions. Then the concentration and purity of the extracted DNA were determined with TBS-380 (Turner BioSystems, Sunnyvale, CA, USA) and NanoDrop 2000 (Thermo-Fisher, Waltham, MA, USA) fluorometry and spectrophotometry respectively, and quality was checked by 1% agarose gel electrophoresis. Paired-end library was constructed after fragmented DNA into an average size of about 300 bp using focused ultrasonication (Covaris M220, Woburn, MA, USA). Then the paired-end sequencing was performed on an Illumina NovaSeq sequencer (Illumina Inc., San Diego, CA, USA). Raw fastq data were quality filtered based on Sickle (https://github.com/najoshi/sickle), then low-quality reads (length < 50 bp, or quality value < 20, or having N bases) were removed. Reads were aligned to the human genome by BWA (http://bio-bwa.sourceforge.net), and any hit associated with the reads and their mated reads were removed. Metagenomics data were assembled using MEGAHIT, and contigs with length ≥ 300 bp were selected as the final assembled result. Open reading frames (ORFs) from each assembled contig were predicted using MetaGene [19]. All predicted genes with a 95% sequence identity were clustered using CD-HIT [20]. Reads after quality control were mapped to the representative sequences with 95% identity using SOAPaligner (http://soap.genomics.org.cn/).

### Annotation and initial analysis

The non-redundant gene sets were aligning CAZymes database (http://www.cazy.org/) based on hmmscan (hmmer 3.0), and the cutoff e-value is set to 1e^-5^. Gene expression was reflected by the RPKM value:

$${RPK}{M}_{i}=\frac{{R}_{i}\times {10}^{6}}{{L}_{i}\times {\sum }_{1}^{n}\left({R}_{j}\right)}$$

Where R_i_ is the abundance value of Gene_i_ in a sample (the reads number aligned to Gene_i_), L_i_ represents the nucleotide length of Gene_i_, ${\sum }_{1}^{n}\left({R}_{j}\right)\,$ represents the sum of reads corresponding to all genes in the sample.

### CAZymes analysis

α-diversity analysis including Simpson 1/D, Shannon H’, Menhinick, Margalef and Berger-Parker indexes [21, 22] was conducted and visualized using the vegan and fossil packages in R, respectively. Principal coordinates analysis (PCoA) was used to visually evaluate the overall difference and similarity of CAZymes between MDD and HC groups [23]. The permutational multivariate analysis of variance (PERMANOVA) was used to test group differences. The differential CAZymes between the two groups were identified using wilcoxon rank sum test (*p* < 0.05). For all CAZymes, we used random forest analysis to screen variables according to the proportion of importance >1% as diagnostic markers, then a 5-fold cross-validation was performed to verify the reliability [24]. The receiver operating characteristic (ROC) curve was obtained (SPSS V.19.0) for the display of the constructed models, then the area under the ROC curve (AUC) was used to designate the ROC effect. Co-occurrence among CAZymes was calculated based on the RPKM by pearson’s correlation coefficient (*p* < 0.05). The network layout was calculated and visualized using a circular layout by the Cytoscape software (version 3.1.1).

### Longitudinal analysis

We enrolled 45 patients undergoing pharmacological treatment and performed long-term follow-up biweekly, and assessed the patients with clinical scores to verify the efficacy of antidepressants(Table. S2). We used a linear mixed-effects model to examine the longitudinal association of gut CAZymes with disease severity (HAMD and QIDS), adjusted for the demographic (age, gender and educational background), anthropometric (BMI), times of depressive episode and medication. Associations were expressed as the difference in HAMD or QIDS score (in SD units) per SD difference in each CAZymes, and *p*-value < 0.05 was considered statistically significant.

## Results

### Demographic and clinical data of the recruited subjects

In this study, gut metagenome data from 311 participants were obtained from our previous studies. All patients with MDD (*n* = 118) were unmedicated at baseline, and there was no statistic difference of demographic characteristics in gender, age or BMI between two groups (Table. S1). In addition, an independent validation set (*n* = 75), whose clinical characteristics did not exactly match, was adopted to verify the diagnostic generalizability of CAZymes markers. In addition, 45 MDD subjects were included in the longitudinal follow-up at 3 time points. The clinical scores were used to quantify the efficacy of antidepressants (Table. S2).

### Alternations of CAZymes signatures in MDD

**Fig. 1:**
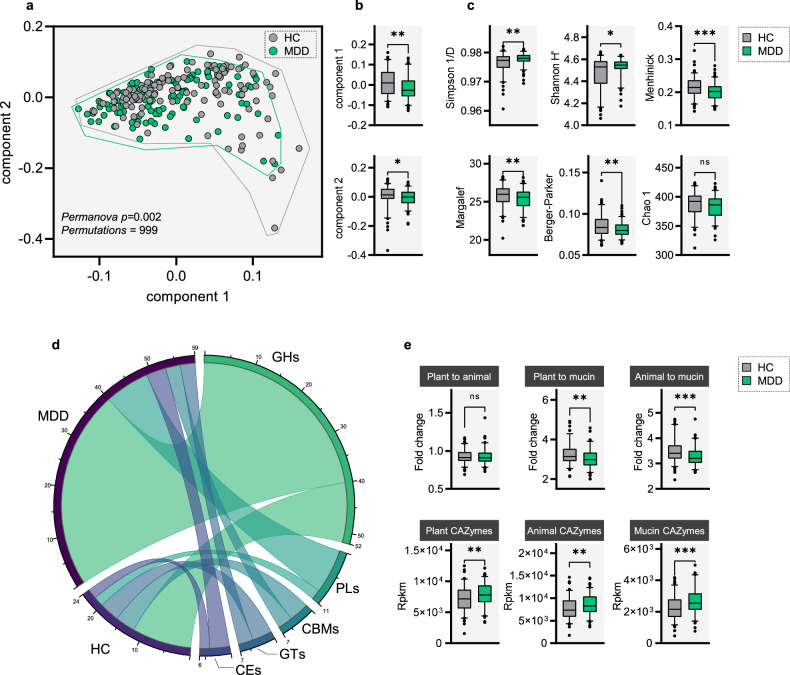
Compositional variation in human gut CAZymes. **a**, **b** The overall characteristics of CAZymes were displayed by Pcoa (bray-curtis distance), internal differences were analyzed with permanova test (permutations = 999, Bonferroni *p* = 0.002). Also, the distance of first and second principal component distance was showed by boxplots (Mann–Whitney *U*-test, *p* = 0.004). **c** Difference of 6 gut CAZymes diversity index between MDD and HC. **d** 83 discriminated CAZymes were identified between two groups (Mann–Whitney *U*-test). **e** The fold change of CAZymes related to plant and animal carbohydrate utilization, plant carbohydrate utilization and mucin glycan and animal to plant carbohydrate utilization in the MDD and HC. The boxplots displayed the respective distributions of three types of CAZymes in metagenome data (Mann–Whitney *U*-test, two-sided). Box-Whisker plot, box = 25–75th percentiles, whiskers = 5–95th percentiles, horizontal line in box = median.

Here, we obtained 23578.431 million paired-end reads on average based on shotgun metagenomic sequencing. We detected a total of 518 CAZymes, and then included 456 CAZymes (prevalence > 10%, mean RPKM > 0.01%) for subsequent analysis. Initially, using the multivariate statistical analysis, we sought to explore whether the whole CAZymes signatures of MDD group were significantly different from that in the HC group. Consequently, we observed significant differences of the whole CAZymes signatures between two group based on Pcoa (bray-curtis distance, permutation test, *p* = 0.002, Fig. 1a), and in the first and second principal component (PC1) (Mann–Whitney *U*-test, *p* = 0.004, Fig. 1b). Next, we compared the α-diversity of CAZymes signatures between MDD and HC groups. We compared the α-diversity of CAZymes signatures between MDD and HC groups. We found that patients with MDD accompanied by 2 increased indexes (Simpson 1/D and Shannon H’) and 3 decreased indexes (Menhinick, Margalef, and Berger-Parker), and there is not significant change in 1 index (Chao 1) (Fig. 1c). In addition, using the wilcoxon rank sum test, we totally identified 83 CAZymes responsible for discriminating the MDD and HC groups (Fig. 1d). These differentially expressed CAZymes mainly involved in Glycoside Hydrolases (GHs, MDD vs HC, 47.0% vs 14.5%), Glycosyl Transferases (GTs, MDD vs HC, 3.6% vs 4.8%), Polysaccharide Lyases (PLs, MDD vs HC, 12.0% vs 2.4%), Carbohydrate Esterases (CEs, MDD vs HC, 4.8% vs 2.4%) and Carbohydrate-Binding Module (CBMs, MDD vs HC, 3.6% vs 4.8%). Our results showed that the utilization of plant and animal carbohydrates in MDD and HC may be in a balance state. Our results showed that compared with HC, the utilization of carbohydrates of MDD may be unbalanced. it is obviously that the MDD microbiota shows a higher preference for plant, animal and mucin utilization. However, the utilization capacity of plants and animal is lower than that of mucin (Fig. 1e).

### Diagnostic gut CAZymes markers for MDD

**Fig. 2:**
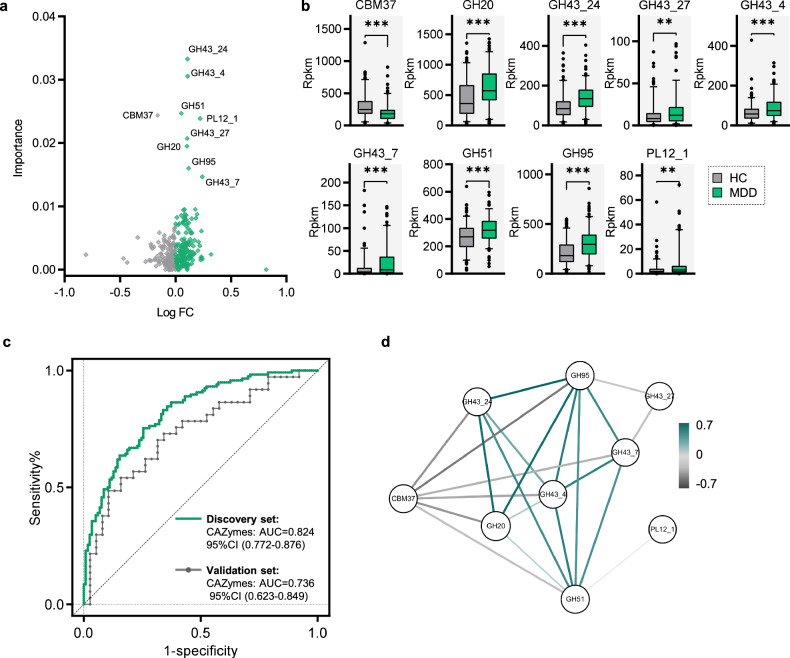
Gut CAZymes features can distinguish MDD and HC. Using random forest model, 9 CAZymes were identified with importance score >1%. **a** Volcano plot for differential CAZymes markers. Significantly regulated metabolites between groups determined by fold change and value of *p* (FC > | 1.5 | , *p* < 0.05). Gray dots re*p*resent increased CAZymes in HC; green dots represent increased CAZymes in MDD. **b** The box plots showed the differences of 9 CAZymes markers (Wilcoxon rank-sum test). **c** A random forest model was constructed and displayed by ROC. In the discovery set, individual signature could discriminate the two groups with area under the curve (AUC) at 0.824, the value in brackets is 95% CI. The diagnostic efficiency was confirmed by 5 fold cross validation test (accuracy: 65.24 ± 7.72%). **d** Correlation-based networks of co-occurring MDD-related CAZymes colored by node affiliation, a co-varying cluster was composed of 6 GHs in MDD subjects. A node stands for an CAZyme and a connection (i.e. edge) stands for a significant (pearson’s *r* > 0.2 or <-0.2, *p* < 0.05) pairwise correlation. Size of the nodes represents the rpkm of these variables. Edges between nodes indicate pearson’s positive (green) or negative (gray) correlation, edges thickness indicate range of *p-*value (*p* < 0.05).

A volcano plot with Fold change was applied to identify the specific CAZymes differences between the two groups. The two groups differed in 9 CAZymes markers (Fig. 2a). Using the random forest model, we identified 9 candidate CAZymes markers for MDD. The results showed that 6GHs (GH20, GH43_24, GH43_4, GH51 and GH95) and 1PL (PL12_1) were significantly increased in MDD group relative to HC group, while 1CBM (CBM37) was decreased (Fig. 2b and Table. S3). We found that a marker panel including this 9 CAZymes enabled to effectively discriminate the samples from MDD and HC, yielding an AUC value of 0.824 (95% confidence interval (CI), 0.772–0.876) (Fig. 2c). In addition, the 5-fold cross validation was performed to further test the diagnostic performance. Then, a validation set was used to independently confirm the diagnostic performance of this marker panel. Consequently, we found that the this CAZymes panel could discriminate blinded test samples from the two groups with an AUC of 0.736 (95% CI, 0.623–0.849), respectively (Fig. 2c).

### Co-occurrence network of CAZymes markers

To explore the potential interaction among CAZymes in MDD, pearson correlation analysis was performed. Here, co-occurrence network depicted the associations (pearson *r* < -0.2 or >0.2, *p*-value < 0.05) among the MDD-related CAZymes (Fig. 2d). Interestingly, 6 GHs including (GH43_24, GH43_4, GH43_7, GH95, GH20 and GH51) generated a covarying cluster, and the members within GHs cluster were negatively correlated to CBM37. In addition, a similar clustering pattern was identified after adding HC samples (Fig. S1).

### Predictive CAZymes markers for MDD

**Fig. 3:**
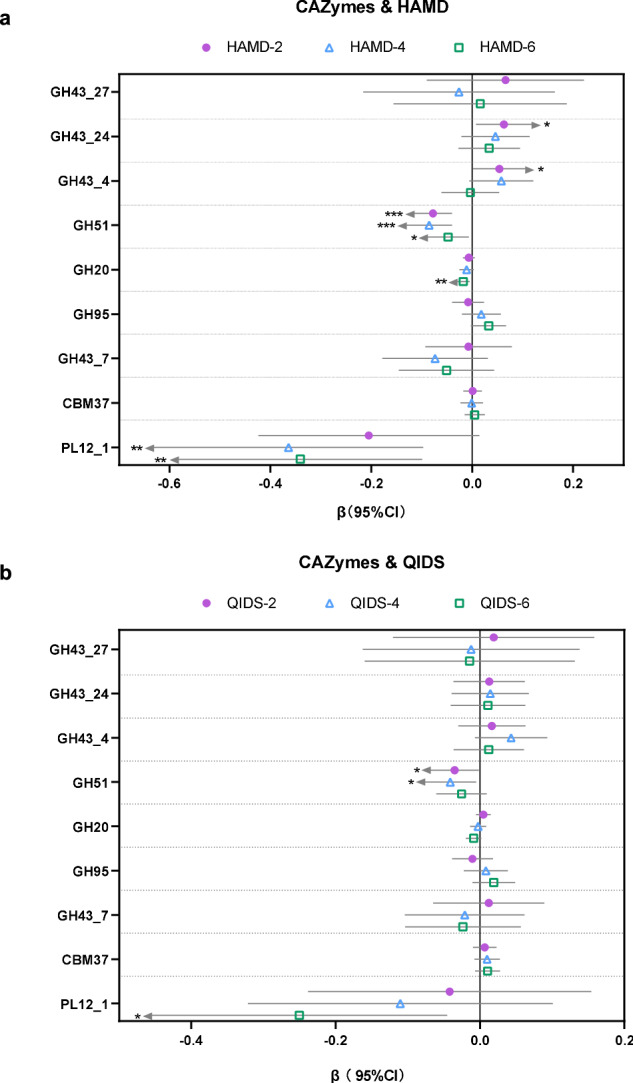
Prospective association of CAZymes markers with severity. **a**, **b** Prospective association of baseline gut CAZymes with HAMD and QIDS score. A total of 45 participants were included in this analysis. Linear mixed-effects model was performed to assess the prospective association of gut CAZymes with the clinical score, adjusting for the demographic (age, gender and educational background), anthropometric (BMI), times of depressive episode and medication. A *p-*value < 0.05 was considered as statistically significant (estimated value β, 95%CI).

Next, 45 patients with MDD who were treated with antidepressants were followed up every 2 weeks (2 weeks, 4 weeks and 6 weeks after treatment) (Fig. S2a). To evaluate the relationship between gut CAZymes and the efficacy of antidepressants, we performed a linear mixed-effects model to examine the longitudinal association of CAZymes markers with MDD disease severity (HAMD, QIDS). A total of 5 gut CAZymes was positively or inversely associated with at least one clinical score (Fig. 3a, b), including 4 GHs (GH43_24, GH43_4, GH20 and GH51) and 1PL (PL12_1). We found that the GH51 (per SD unit) showed a significant inverse association with HAMD (2w: estimated value −0.078, 95% CI: −0.115, −0.040; 4w: estimated value −0.085, 95% CI: −0.131, −0.040; 6w: estimated value -0.048, 95% CI: −0.089, −0.007) and QIDS (2w: estimated value −0.035, 95% CI: −0.069, −0.002; 4w: estimated value −0.041, 95% CI: −0.077, −0.005). Consistently, PL12_1 was also found to be inversely associated with HAMD scores at 4, 6 weeks after antidepressants (4w: estimated value −0.364, 95% CI: −0.631, −0.097; 6w: estimated value -0.341, 95% CI: -0.582, −0.099), and 6 weeks QIDS (6w: estimated value −0.250, 95% CI: −0.455, −0.046). In addition, 2 GHs showed a positive association with 2 week HAMD scores (GH43_24: estimated value 0.063, 95% CI: 0.007, 0.118; GH43_4: estimated value 0.054, 95% CI: 0.002, 0.106). Moreover, GH20 was found to be only related to the 6 week HAMD scores. However, none of GHs or PLs were found to be associated with clinical score at baseline group (Fig. S2b).

## Discussion

Clinical and basic studies have shown the link between gut microbiome and MDD. As the fraction of gut microbiome containing the gene coding for CAZymes, here we charactered how the CAZymes changed in the MDD relative to HC. Carbohydrates can regulate gut microbiota metabolism and maintain resident bacterial populations [25]. Interestingly, we found that MDD was associated with enriched Glycoside Hydrolases and Polysaccharide Lyases. A random forest model constructed by 9 CAZymes could effectively distinguish patients with MDD and HC, then the diagnostic performance of this CAZymes panel was independently verified. Meanwhile, based on linear mixed-effects analysis, we found that 4 CAZymes could predict the therapeutic effects in the patients treated with antidepressants. Our research provides new ideas for the accurate diagnosis and treatment of depression. Based on the finding, modifying dietary patterns to alter CAZyme expression may alleviate the symptoms of MDD. The metagenome data suggest that the gut microbiota of MDD have a greater mucin-utilization capacity relative to HC. It may because of the high concentration of *Bacteroides* in MDD population. In addition, the increased availability of mucin may weaken the mucous layer of the intestinal wall, and some *Bacteroides* can degrade mucin during survival, such as *B. thetaiotaomicron*, *B. fragilis*, as a result, harmful molecules in MDD are more likely to cross the gut and enter the circulatory or endocrine system [26].

Alternations of gut microbiome were closely related to depression. In the past, we and other teams focused on exploring how the gut bacteria and viruses changed in MDD [15, 27, 28]. In addition, using fecal bacteria transplantation and probiotic intervention, preliminary evidences showed that disturbances of gut microbiome may attribute to development of depression by regulating the MGB axis’ metabolism [29, 30].These studies lay a foundation for further study of the role of gut microbiome in depression. Here, we identified the differentially expressed CAZymes in the patients with MDD relative to HC, hoping to further understand the function of gut microbiome in depression from this new point. Here, we found MDD is substantially linked with alternations of CAZymes relative to HC. Compared to HC, the MDD was characterized by enriched Glycoside Hydrolases and Polysaccharide Lyases. Bacteroidetes have ability to package CAZymes into membrane vesicles and release them into the extracellular environment, enabling other members of subsequent products to have access to available carbohydrates [7]. Since gut microbiota can encode CAZymes [31], dietary fiber can undergo multiple reactions through the action by CAZymes, then producing substances such as SCFAs, which participate in bidirectional communication between the gut and brain [32, 33]. Given Bacteroides, especially *Bacteroides* thetaiotaomicron and *Bacteroides* ovatus, dedicate about 6% of their genomes to encode these two CAZymes [8]. These results suggesting that alternations of *Bacteroides* species was a hallmark of MDD, which was consistent with our previous studies [15, 16].

In addition, we found that the CAZymes has potential diagnostic and predictive value in MDD. For example, CAZymes maker panel enable discriminating the MDD and HC with the values of 0.824, suggesting that it has potential clinical transformation value. Clinically, predicting the efficacy of antidepressants remains challenging prior to antidepressant medication, thus inevitably, the trial and error of experience lies in the replacement of new drugs. Here, we found that markers belonging to Glycoside Hydrolases increased in baseline MDD relative to HC. Interestingly, the baseline level of GH51 was negatively correlated with HAMD (2, 4 and 6 weeks) and QIDS (2 and 4 weeks) scores. Consistently, PL12_1 was also negatively correlated with these two clinical scores of 6 weeks. An enzyme (α-L-arabinofuranosidase) in the GH51 family, encoded by two dominant *Bacteroides* (B. ovatus V975 and B. thetaiotaomicron VPI-5482) [34, 35]. Another CAZyme (PL12_1) contains 2 types of heparin lyase, which were found to be encoded by B. stercoris HJ-15 and B. thetaiotaomicron VPI-5482 [36, 37]. These results suggesting alternations between drugs and biological functions of *Bacteroides* involved in antidepressant effects. Given CAZymes could be detected in feces, it lays a foundation for the development of noninvasive diagnostic and predictive kits.

Our study has the following shortcomings: (1) although a relatively large sample size is used to identify CAZymes as candidate markers, this result still needs to be independently verified by a large multi-center sample; (2) In depth study of the function of CAZymes is expected to further understand the roles of gut microbiome in the pathophysiological mechanisms of MDD; (3) Antidepressants can affect the composition and function of the gut microbiome [38], mediating the expression of microbial-encoded CAZymes. However, gut microbiota participate in the metabolic reactions and transformations of duloxetine and clonazepam, leading to individual differences in drug efficacy [39, 40]. Additionally, some CAZymes, such as UDP-glucuronosyltransferase, can be replaced by a single drug molecule and covalently link the β-glucuronide moiety to an available group, reducing drug availability [7]. Overall, the interactive mechanisms between gut microbiota and CAZymes require further exploration; (4) Since CAZymes are mainly involved in the digestion of polysaccharides, it is also worth exploring how to formulate an individualized diet which is more suitable for the microecological health of MDD; (5) Future researches to explore whether intervention of CAZymes has potential therapeutic effects in MDD are required.

In this study, using well-characterized cross-section and longitudinal clinical samples, we provided evidences that MDD was associated with disturbances of gut CAZymes. Moreover, we found that the CAZymes markers can be used to diagnose the patients with MDD and predict antidepressant effects. Our findings suggest that alternations of CAZymes may be a new entry point to understand the roles of gut microbiome in the development of MDD.

### Supplementary information

Supplemental Figure.1

Supplemental Figure.2

Supplemental Table.1

Supplemental Table.2

Supplemental Table.3

## Data Availability

The metagenomic sequencing data were deposited in the China National GeneBank DataBase (CNGBdb) (https://db.cngb.org/; project ID: CNP0001162).
