# Supplementary material for: Gut microbial CAZymes markers for depression
Source: Transl Psychiatry. 2024 Mar 5;14:135. doi: 10.1038/s41398-024-02850-x (PMC10914822; doi:10.1038/s41398-024-02850-x)
Supplement: Supplementary file 1 — Supplemental Figure.1 [file 41398_2024_2850_MOESM1_ESM.pdf]

Supplemental Figure 1

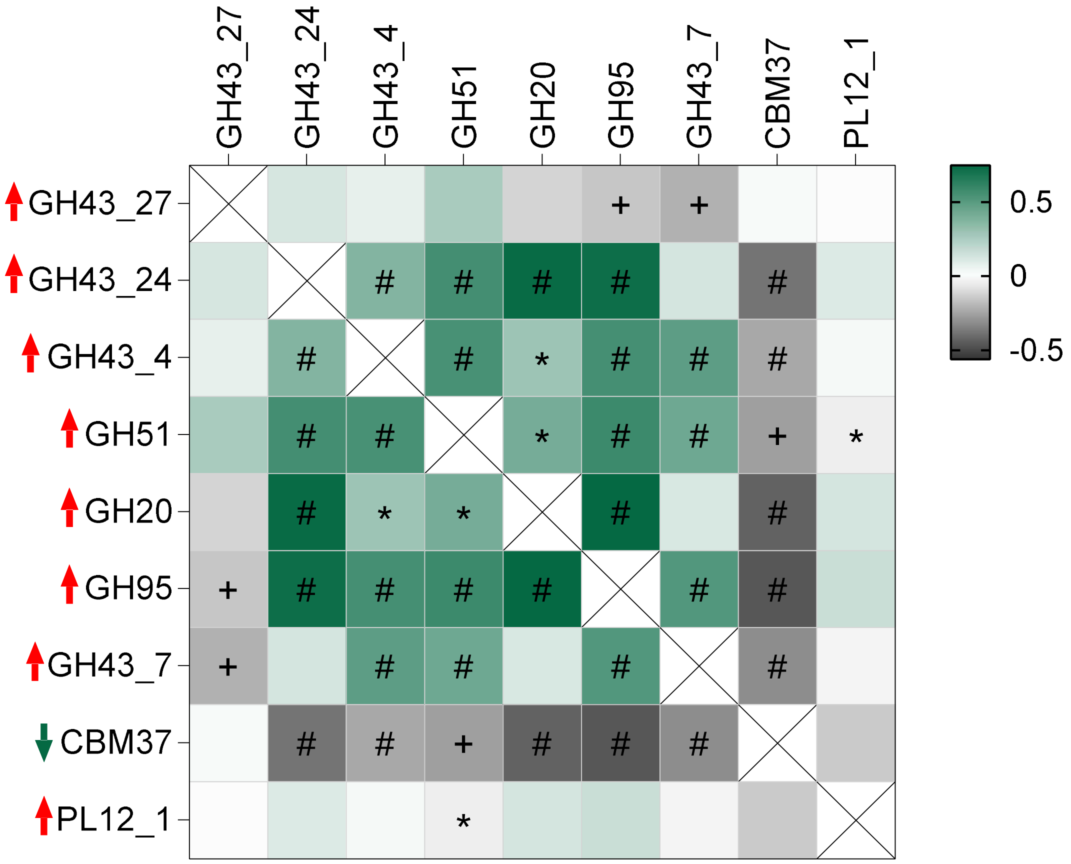

Supplemental Figure 1. **Co-occurrence patterns among MDD-related CAZymes.** Association between CAZymes based on pearson analysis (MDD, n=118, HC, n=118; * p<0.05, + p<0.01, # p<0.001), red arrows indicated an increase in MDD related to HC, while green arrows indicated a decrease in MDD.
