# Supplementary material for: Gut microbial CAZymes markers for depression
Source: Transl Psychiatry. 2024 Mar 5;14:135. doi: 10.1038/s41398-024-02850-x (PMC10914822; doi:10.1038/s41398-024-02850-x)
Supplement: Supplementary file 2 — Supplemental Figure.2 [file 41398_2024_2850_MOESM2_ESM.pdf]

Supplemental Figure 2

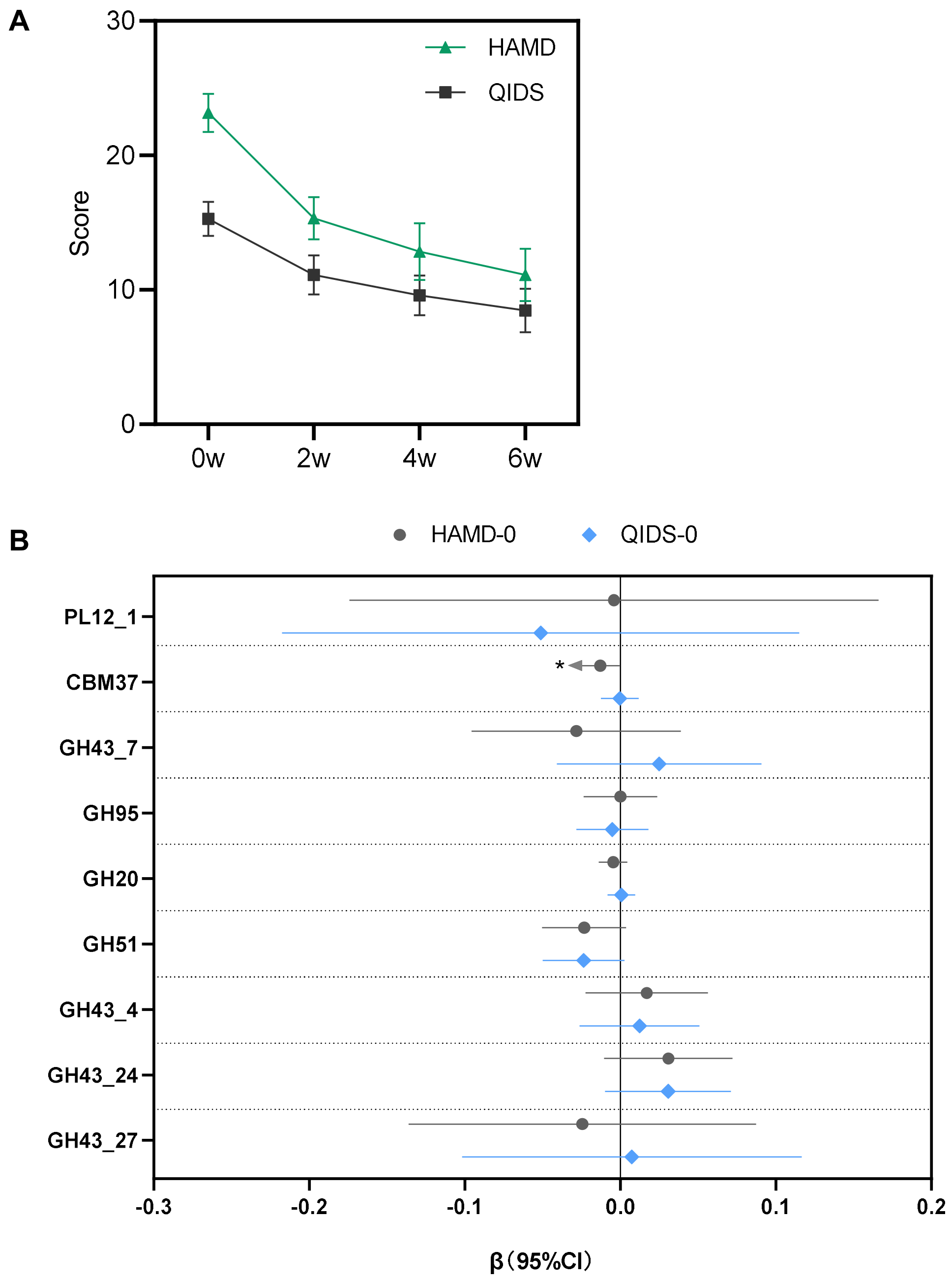

Supplemental Figure 2. a. Trend of clinical scores after taking antidepressants, bar represents 95% CI. **b**. Linear regression model results based on baseline CAZymes and clinical scores.
